# Saccharine

**Published:** 1888-10

**Authors:** 


### SACCHARINE.

In a recent interview with Dr. Constantine Fahlberg, the discoverer of the new sugar extracted from coal tar, he said concerning his relation to this new product: "I had worked a long time upon the compound radicals and substitution products of coal tar, and had made a number of scientific discoveries that are, so far as I know, of no commercial value. One evening I was so interested in my laboratory that I forgot about supper until quite late, and then rushed off for a meal without stopping to wash my hands. I sat down, broke a piece of bread, and put it to my lips. It tasted unspeakably sweet. I did not ask why it was so, probably because I thought it was some cake or sweetmeat. I rinsed my mouth with water, and dried my mustache with my napkin, when, to my surprise, the napkin tasted sweeter than the bread. Then I was puzzled. I again raised my goblet, and, as fortune would have it, applied my mouth where my fingers had touched it before. The water seemed syrup. It flashed upon me that I was the cause of the singular universal sweetness, and I accordingly tasted the end of my thumb, and found that it surpassed any confectionery I had ever eaten. I saw the whole thing at a glance. I had discovered or made some coal tar substance with out-sugared sugar. I dropped my dinner and ran back to the laboratory. There in my excitement, I tasted the contents of every beaker and evaporating dish on the table. Luckily for me, none contained any corrosive or poisonous liquid.

"One of them contained an impure solution of saccharine. On this I worked then for weeks and months until I had determined its chemical composition, its characteristics and reactions, and the best modes of making it scientifically and commercially.

When I first published my researches, some people laughed as if it were a scientific joke, others, of a more skeptical turn, doubted the discovery and the discoverer, and still others proclaimed the work as being of no practical value.

"When the public first saw saccharine, however, everything changed. The entire press, European and American, described me and my sugar in a way that may have been edifying, but was simply amusing to me. And then came letters. My mail run as high as sixty a day. People wanting samples of saccharine, my autograph, or my opinion on chemical problems, desiring to become my partner, to buy my discovery, to be my agent, to enter my laboratory, and the like,"

